# Northward Range Expansion of Water Deer in Northeast Asia: Direct Evidence and Management Implications

**DOI:** 10.3390/ani12111392

**Published:** 2022-05-28

**Authors:** Ying Li, Jee Hyun Kim, Hailong Li, Yuxi Peng, Min Chen, Weihong Zhu, Puneet Pandey, Gleb Sedash, Tianming Wang, Yury Darman, Hang Lee

**Affiliations:** 1 Tiger & Leopard Conservation Fund in Korea (KTLCF), and Research Institute for Veterinary Science, College of Veterinary Medicine, Seoul National University, Seoul 08826, Korea; shadowlee@snu.ac.kr (Y.L.); kjhsonic@hanmail.net (J.H.K.); puneet.pandey09@snu.ac.kr (P.P.); 2 Geography Department, College of Geography and Ocean Science, Yanbian University, Yanji 133002, China; hai3456@snu.ac.kr (H.L.); 2184231366a@gmail.com (Y.P.); whzhu@ybu.edu.cn (W.Z.); 3 National Forestry and Grassland Administration Key Laboratory for Conservation Ecology in the Northeast Tiger and Leopard National Park, Beijing 100875, China; 4 School of Life Sciences, Institute of Eco-Chongming, East China Normal University, Shanghai 200062, China; mchen@bio.ecnu.edu.cn; 5 Land of the Leopard National Park, 692723 Vladivostok, Russia; sedash@leopard-land.ru; 6 Ministry of Education Key Laboratory for Biodiversity Science and Engineering, and College of Life Sciences, Beijing Normal University, Beijing 100875, China; 7 Amur Branch of World-Wide Fund for Nature, 690003 Vladivostok, Russia

**Keywords:** water deer, *Hydropotes inermis*, camera traps, range expansion, mtDNA

## Abstract

**Simple Summary:**

The water deer *Hydropotes inermis*, one of the most primitive members of Cervidae, is classified as vulnerable by the IUCN Red List in their native range, having declined drastically in recent years. In this study, we illustrated a northern extension of water deer using the current evidence, and assessed the status, phylogeny, and genetic ancestry of the newly recorded population. Our results showed that water deer had expanded to the Northeast China and the Russian Far East, where there had previously been no record of this species; thus, this could be a genuine range expansion rather than simply an expansion of the known range. A genetic investigation indicated that the expanding population had a close phylogenetic affinity with Korean water deer. The likely migration route and causes of the species’ distribution range expansion are discussed.

**Abstract:**

Given current anthropogenic pressures and climate change, wildlife range expansion offers a second chance for species conservation. The water deer *Hydropotes inermis* is a native to China and the Korean peninsula, but populations in North Korea and mainland China have declined drastically in recent years. However, the range of this species appears to be rapidly expanding northward. In this study, we employed camera traps and molecular technology to assess the status, phylogeny, and genetic ancestry of the newly recorded population. Our results showed an ongoing northward expansion of water deer, reaching at least 500 km from their historical distribution limit. We provided updated information on this species’ geographical distribution in Northeast China and the Russian Far East. Based on historical survey data before the 1990s, there had previously been no record of this species in these two regions; thus, this could be a genuine range expansion rather than simply an expansion of the known range. A genetic investigation based on mitochondrial DNA indicated that the expanding population had a close phylogenetic affinity with Korean water deer. The likely migration route and causes of the species’ distribution range expansion are discussed. We recommend revising the range of water deer in the IUCN Red List to facilitate the effective conservation and management of this threatened species, especially in new locations.

## 1. Introduction

The water deer (*Hydropotes inermis* Swinhoe, 1870), one of the most primitive members of Cervidae, is classified as “Vulnerable” by the IUCN Red List in its native range, having declined drastically in recent years, primarily through habitat loss and degradation, and extensive illegal hunting [1,2]. This species was once widely distributed in eastern China and the Korean peninsula, reaching as far north as China’s Liaoling Province [3,4,5]. Water deer are solitary and adaptable to a range habitat, and they are mostly found in forest wetlands, meadows, river valleys, and coastal habitats, but also in agricultural land, especially rice paddies [3,6]. This species is characterized by an unusually high fecundity. Female water deer reach sexual maturity at two years of age, and they are well-adapted to the ecological opportunities and risks in a changing environment [2,7]. It is the only extant deer species that lacks antlers, which is thought to result from a secondary loss within Cervidae [8]. Traditionally, two subspecies are recognized and distinguished by their geographic distribution: the Chinese water deer (*H. i. inermis* Swinhoe, 1870) and the Korean water deer (*H. i. argyropus* Heude, 1884) [9,10]. Chinese water deer are an understudied species with fragmented distributions in Southeast China; today, the known northernmost part of their range is in the coastal area of Jiangsu Province [4,11,12].

Recently, water deer were discovered in Jilin Province of China [13,14]. This was the first record of water deer in Northeast China in the last seventy years. Historically, there existed records of water deer in Russia. In 2019, a camera trap took the first photograph of water deer in Southwest Primorsky Krai. Additionally, hunters killed a deer originally thought to be a musk deer (*Moschus moschiferus*), but it was later identified as a water deer by experts based on photographs and skull measurements [15]. Based on this evidence, the water deer was officially announced as a new species on the Russian mammal list [16].

In addition, from a conservation point of view, wildlife range shift provides an important opportunity for conservation efforts. Current conservation efforts for this species are critically dependent on an in-depth understanding of its ecology and genetics, but comprehensive and reliable information on expanded population ranges and genetic connectivity with existing subspecies is lacking. Here, we used camera-trap data and molecular data, along with other survey evidence, to assess the species identification, distribution, and genetic relationship of two water deer species to clarify the origin of the population. We hypothesized that this population distribution was not simply an expansion of the known range, and this deer shared a close phylogenetic relationship with the Korean subspecies. We believe the new population occurred by migration/translocation from the Korean peninsula or mainland China. Our findings of the water deer provide insight into the confirmed species identification, range, genetic relationship, and ongoing management of them. Since our sample size was limited, further sampling of the discovered population will be necessary to confirm its genetic status and relationship to other water deer populations.

## 2. Materials and Methods

### 2.1. Study Area

**Figure 1:**
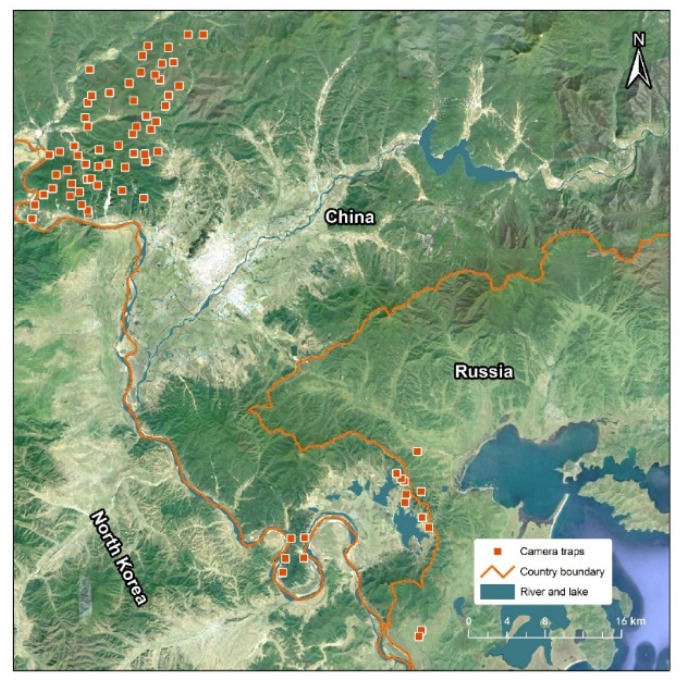
Map of our camera-trapping study area in Northeast China, adjacent to the Land of Leopard National Park in Russia.

A camera-trapping survey for this study was conducted in Hunchun, Jilin Province, China, and in Southwest Primorsky Krai region of the Russian Far East, located in the transboundary area of the downstream Tumen River (Figure 1). The Tumen River marks the border between China and North Korea; further downstream, a 15 km area marks the boundary between Russia and North Korea. The area is a temperate coniferous broadleaved mixed forest with a mild climate and high biodiversity, inhabited by many important flagship species or subspecies of Northeast Asia, such as the Amur tiger (*Panthera tigris altaica*) and Amur leopard (*P. pardus orientalis*), red-crowned crane (*Grus japonensis*), white-naped crane (*Antigone vipio*), white-tailed eagle (*Haliaeetus albicilla*), and chum salmon (*Oncorhynchus keta*) [14].

### 2.2. Camera-Trap Survey

Camera-trap stations were selected considering water deer biology, habitat preference, and activity pattern to enhance capture probability [3,6]. In total, 74 camera stations were established (Figure 1). In China, 12 camera stations were established in the Jingxin wetland area downstream of the Tumen River basin from January to April 2020, and 57 stations were established along the Mijiang River area, a tributary of the Tumen River, from March 2019 to July 2021. In Russia, 3 camera stations were established in the Tesnaya River wetland near Peter the Great Bay from July to November 2019, and 2 stations were established in the Karasik wetland area from March to June 2020. Camera traps (LTL 6210M, Shenzhen, China) were fastened to trees approximately 40–80 cm above the ground and programmed to record 24 h/day with a 1 min interval between consecutive 15 s videos. We report the number of detections for each species. Water deer identification followed morphological characteristics, which can differentiate roe deer (*Capreolus pygargus*) by their tails and teeth (water deer have small tails, and male water deer have long canine teeth, which roe deer do not have); at the same time, water deer can be differentiated from musk deer by their tails and fur color (water deer have short tails and a light brown fur color compared to musk deer) [5,12]. To reduce inflated counts caused by repeated detections of the same event, only one record of a species at a trap site was recorded per 0.5 h.

### 2.3. Other Occurrence Records

During the field survey, we opportunistically collected water deer occurrence data, such as hoofprints and scat, based on spotting individuals and determining GPS locations. Field signs were safely attributed to species based on at least two experts. Occurrence records were also gathered from the literature [13,14,15,16,17], evidence from roadkill, hunts, photographic, and published technical reports. This information was extracted to provide a better understanding of the new and expanding water deer range.

According to Zhang [18], we obtained water deer presence records to determine the historical distribution of the Chinese water deer (Figure S1). Data included: (1) early publications or literature since the 1920s, (2) information on faunal investigations and records of specimens, which were contributed by integrated scientific expeditions of Academia Sinica since the 1950s, and (3) publications about mammals at the provincial level before the 1990s.

### 2.4. Sample and Molecular Analysis

We performed mitochondrial DNA analysis to understand the phylogeny and genetic ancestry of water deer in Northeast China and Russia. DNA was extracted from five tissue samples (China: 2; Russia: 3) belonging to individuals that had either died naturally, in road accidents, or were killed by poachers (Table S1). The samples were provided by the national park administrations, and necessary permissions were granted for genetic investigation by the responsible authorities. DNA was extracted using the Qiagen Blood and Tissue DNA extraction kit following the recommended protocols and precautions. We amplified a partial fragment of the mitochondrial cytochrome *b* gene [19] and D-loop sequences [20]. Positive amplifications were sequenced (in both directions) using the Bigdye terminator v3.1 cycle sequencing kit (Applied Biosystems, Waltham, MA, USA) and 3730 genetic analyzer (Applied Biosystems, USA). Genius Prime software was used to check the sequence quality, editing, and alignment. The species identity of each of the analyzed samples was reconfirmed using NCBI BLAST. The phylogenetic position of water deer samples collected from the newly expanded range in Northeast China and the Russian Far East was verified in two steps: (1) among other deer species [21] using the cytochrome *b* gene and (2) within water deer subspecies using D-loop sequences [10,20,22].

## 3. Results

In the downstream Tumen area during the period of 2019 to 2021, we recorded 11,066 detections of 19 mammal species over 32,374 trap days at 74 camera stations (Table 1). We detected 19 mammals in the research area, including water deer, roe deer, wild boar (*Sus scrofa*), sika deer (*Cervus nippon*), Amur leopard *(Panthera pardus orientalis*), Amur tiger (*Panthera tigris altaica*), Asian black bear (*Ursus thibetanus*), brown bear (*Ursus arctos*), red fox (*Vulpes vulpes*), raccoon dog (*Nyctereutes procyonoides*), Siberian weasel (*Mustela sibirica*), Eurasian otter (*Lutra lutra*), Siberian chipmunk (*Tamias sibiricus*), Eurasian red squirrel (*Sciurus vulgaris*), Amur hedgehog (*Erinaceus amurensis*), yellow-throated marten (*Martes flavigula*), leopard cat (*Prionailurus bengalensis euptilurua*), Manchurian hare (*Lepus mandshuricus*), and Asian badger (*Meles leucurus*). Total detections varied across the study sites, ranging from 1 detection of a brown bear (*Ursus arctos*) and Eurasian otter (*Lutra lutra*), respectively, to 4078 of roe deer.

**Figure 2:**
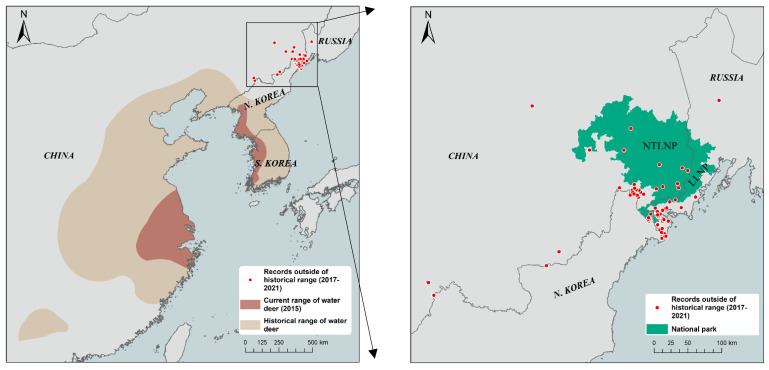
Range of the water deer. Historical range according to Whitehead (1993) [23] is shown in light tan, and current range in China and Korea peninsula according to the IUCN Red List is shown in dark tan [1], while red dots indicate individual recordings of water deer outside of the historical range. Green area (right) indicates the Northeast Tiger and Leopard National Park of China (NTLNP) and the Land of Leopard National Park of Russia (LLNP).

**Figure 3:**
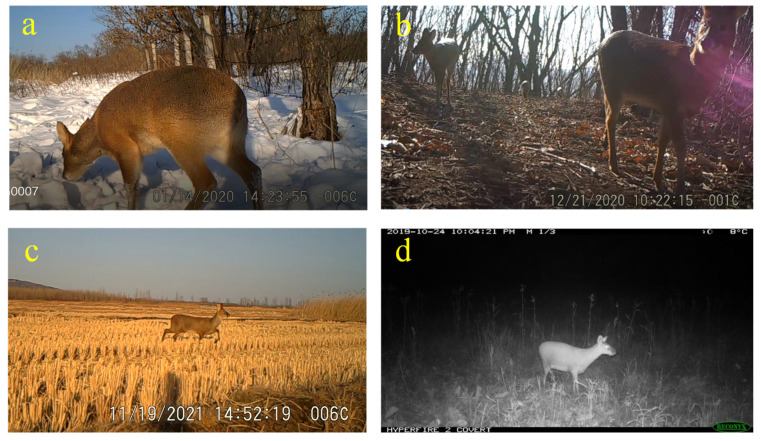
Photographs of water deer captured by camera-traps in the expansion areas in China and Russia. (**a**) A male in forest wetlands: 14 January 2020, (**b**) a female with fawns in the deciduous forests: 12 December 2020, (**c**) a male in rice paddies: 19 November 2021, and (**d**) a male in seasonal swamps: 24 October 2019.

We strictly followed the morphology identification characteristics to differentiate water deer from other deer species. A total of 83 independent detections of water deer were captured at 32% (*n* = 24) of the camera stations. In the Mijiang River area, naïve occupancy ranged from 16% to 19% during the period. Water deer with fawns were also detected in the wild, and the deer occurred in diversified habitats, including forest wetlands, swamps, and croplands (Figure 2 and Figure 3). In addition to our camera-trapping data, 95 other ecology data occurrence records were obtained in the new expansion area from 2017 to 2021, including 37 from the photographic evidence, 53 from the field survey, 2 from the roadkill, and 3 from hunted individuals. We used the overall records to update the range of water deer (Figure 2), and this species has recently expanded beyond their known geographical distribution (Figure S1).

**Figure 4:**
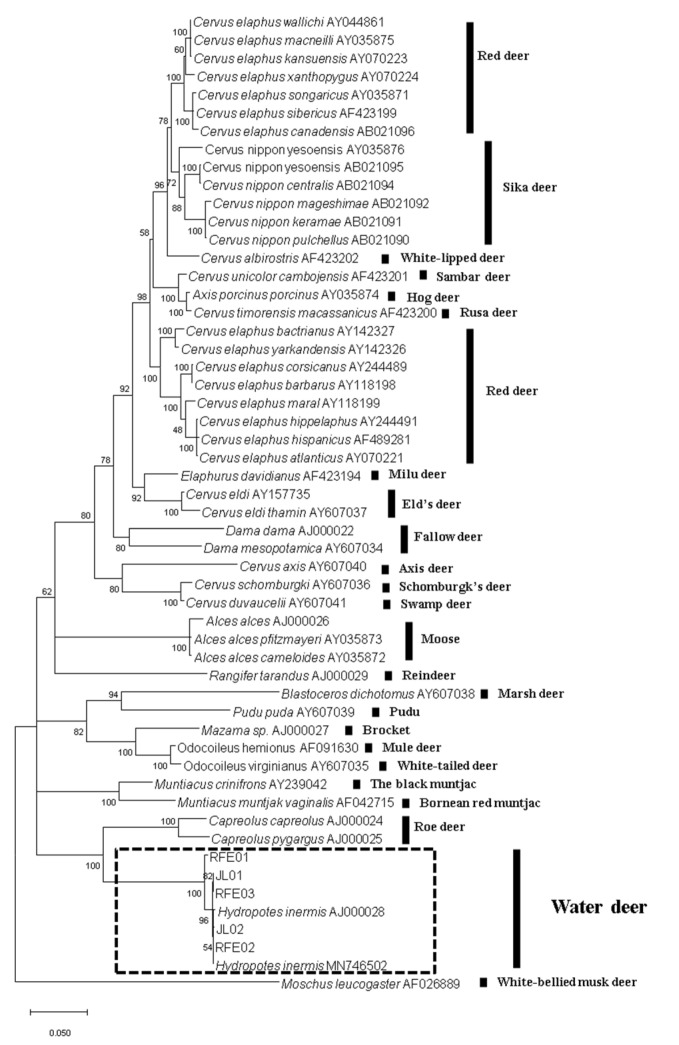
Species identification using a genetic tool. Maximum likelihood tree using cytochrome *b* sequences from deer species.

The consensus sequences of 1140 bp for cytochrome *b* and 822 bp for the D-loop were obtained for the analyzed samples. NCBI BLAST suggested that the tested samples belonged to water deer. Furthermore, the phylogenetic analysis with cytochrome *b* gene sequences of 49 deer species reconfirmed the species identity (Figure 4). A phylogenetic tree based on mitochondrial D-loop sequences suggested the existence of two broad subgroups (Figure S2). We reported four haplotypes in the five water deer samples, of which two were novel (samples JL1 and RFE03). The remaining three haplotypes had previously been described for South Korean water deer [21]. Altogether, the tested samples and other water deer sequences represented a total of 22 haplotypes (H1 to H22; Figure S3). Haplotype 21 (H21, South Korean water deer haplotype) was reported in two samples: one each from Northeast China (JL02) and the Russian Far East (RFE01) (Figure S3).

## 4. Discussion

In this study, we illustrated the increased distribution of water deer using current evidence. Our findings provide new information on the species’ geographical distribution, which could represent a genuine range extension, rather than a novel finding of range that has been occupied all along. Our findings will help inform conservation strategies for this threatened animal. Water deer have been sporadically spotted by local forestry workers and border guards in northeast China and in the China–Russia transboundary region since 2010.

In China, water deer reappeared in their historical range along the Yalu River, and photographs were first taken in the Jilin Baishan Musk Deer National Nature Reserve in December 2017 (41.7972 E, 126.4711 N) [13]. In November 2017, a picture of a previously unseen deer was taken by camera-traps in Southeast Heilongjiang Province (Dongjingcheng Forestry Bureau, 44.1327 N, 128.3789 E), which is significantly northward of the known sightings of this ungulate in China, approximately 300 km from the Baishan Reserve in Jilin Province. In Russia, the northernmost known record from a hunted individual occurred in the Mikhailovskiy district of the Primorsky Krai in 2014 (44.1135 N, 131.8284 E), and water deer invaded from downstream of the Tumen River to the north of the Primorsky Krai (approximately 1000 km^2^) [15,16]. In the historical research for several decades by different organizations in Jilin and Heilongjiang provinces, the new range of the species was not included (Figure S1). The frequent reports on water deer roadkill and monitoring have only happened in the last ten years in Jilin and the Russian Far East. The range expansion is the most likely reason for the species’ appearance in the area. Thus, to the best of our knowledge, we have documented the northernmost range of the water deer in Northeast Asia, reaching at least 500 km from their historical distribution limit. Our data suggest that water deer populations may be recovering within their historical range, as well as vastly increasing their population and distribution beyond their current range. We recommend extensive field surveys, collecting ecological data and photographic evidence, to clarify the distribution limits, population size, and preferred habitat of water deer, as well as long-term monitoring to assess their dispersal speed and persistence.

Species range expansions, or appearance in new locations, can occur through human-mediated reintroductions or translocations, climate change-driven range shift via migration and dispersal, or anthropogenic habitat changes, etc. As there are no official records of water deer translocation in China in recent decades, the anthropogenic movement of water deer from Southeast China to a new range appears implausible. Even though we cannot rule out the undetected or illegal translocation of animals by unidentified individuals, there does not appear to be obvious incentives for unregulated live wild animal translocation. Similarly, due to the greater geographical distance and urbanization, range expansion via the natural migration of water deer from Southeast China is also highly unlikely.

Natural range expansion from North Korea to Northeast China and Russia is possible due to habitat connectivity. There is little available information on the historical and current distribution of water deer in North Korea. Existing species distribution maps indicate that water deer are distributed along the west coast of North Korea [1]. Between the late 1950s and the late 1960s, the North Korean government relocated water deer from the west coast to the east coast [24]. If the translocated population survived and spread to the north, it could have served as a source population for the newly expanded range. Local rangers confirmed this potential water deer movement in January 2020, when they discovered numerous tracks in the snow of water deer crossing the Tumen River near its mouth from North Korea to Russia (according to a questionnaire survey). Additionally, in May 2019, a water deer was reportedly wounded in a road accident around the Tumen River near the North Korea border in Russia [17]. The transboundary population is genetically comprised of various lineages and haplotype groups (Figures S2 and S3), and its composition is very similar to that of the South Korean water deer population [6]. As there was not a geographical or ecological barrier between North and South Korea before the Korean War, we can assume that the genetic composition of the North Korean water deer population is similar to that of the South Korean population. Thus, the similarity in genetic composition between the transboundary population and the South Korean population suggests that the two populations are related and supports the North Korean origin of the newly established population.

Water deer are unusual in that their primitive adaptations are normally associated with warm climates [25]. However, this species is adapted to cold-temperate seasonal climates with frost and snow. In Northeast Asia, the average annual temperature rose by approximately 0.2–0.8° cover the 10 years from 2010 to 2019 compared to the long-term average annual temperature (1981–2010) (The National Atlas of Korea II, http://www.ngii.go.kr accessed on 21 April 2022). Since water deer prefer lowlands [2], a warmer climate may make them disperse to higher latitudes and altitudes. However, more research is needed to determine the role of climate change in promoting the northward spread of this species and to identify potential corridors.

We found a relatively high number of haplotypes (four in five samples) in the Northeast China–Russia population, despite our limited sampling. Some of these haplotypes were similar to those previously reported from South Korea, but two of them were novel. Thus, it is highly likely that the water deer in the newly established range in Northeast China and Russia may have originated from North Korea or represent the historical native population, but this requires further validation with increased genetic sampling and the analysis of more genes. The genetic evidence presented here emphasizes the importance of the extensive sampling of water deer across their current range to update information on their origin, phylogenetic relationships, and genetic variation, which is suggested by Schilling and Rössner [2]. Future research should also include a comprehensive ecological and molecular assessment using high-throughput sequencing and a species distribution model to determine whether the expanding population is a recent undiscovered expanding population in China or spread from the Korean peninsula.

The expansion of water deer will enrich the transboundary ecosystem, and they may serve as an additional prey for predators (such as endangered tigers and leopards), especially in China’s Northeast Tiger and Leopard National Park and the neighboring Land of Leopard National Park in Russia. We observed that there was broad sympatry among the three deer over a large area (Table 1). However, the negative consequences of the new expanded deer and how the animal interacts with the species in its family—roe deer and sika deer—and affects local plants require long-term monitoring. This is because water deer have a high fecundity compared to other deer species: female deer can breed three to four calves on average each year, with a maximum record of seven calves at a time, and calves achieve sexual maturity after one year [26].

## 5. Conclusions

The northward expansion of water deer was confirmed by the evidence of camera-trapping and genetic tools. By discussing the habitat connectivity and haplotype similarities, we argue that this species very likely expanded from North Korea. We recommend considering this water deer population as unique, with no attempts at individual reintroduction or translocation from China or the Korean peninsula until a thorough investigation into the population’s origin has been completed. As the northward spread of this deer may be unstoppable, collaboration between scientists and managing institutions across all water deer range countries is critical for a full assessment of the population status and a landscape conservation plan. Additionally, we propose amending the IUCN Red List distribution range for water deer to facilitate the evidence-based management and conservation of these populations in a human-dominated landscape.

## Figures and Tables

**Table 1:** A list of each species camera-trapped in different study regions, including the number of independent detections, number of camera-traps, and working days.

	Tesnaya	Karasik	Jingxin	Mijiang	Total
Number of camera-traps	3	2	12	57	74
Working days	294	90	945	31,045	32,374
Water deer (*Hydropotes inermis*)	3	3	9	68	83
Roe deer (*Capreolus pygargus*)	141	22	162	3753	4078
Wild boar (*Sus scrofa*)	190		284	1536	2010
Sika deer (*Cervus nippon*)	42		1	176	219
Amur leopard (*Panthera pardus orientalis*)	1	1		21	23
Amur tiger (*Panthera tigris altaica*)	1			1	2
Asian black bear (*Ursus thibetanus*)				7	7
Brown bear (*Ursus arctos*)	1				1
Red fox (*Vulpes vulpes*)			12	850	862
Raccoon dog (*Nyctereutes procyonoides*)			17	1909	1926
Siberian weasel (*Mustela sibirica*)			3	116	119
Eurasian otter (*Lutra lutra*)				1	1
Siberian chipmunk (*Tamias sibiricus*)				10	10
Eurasian red squirrel (*Sciurus vulgaris*)				21	21
Amur hedgehog (*Erinaceus amurensis*)				61	61
Yellow-throated marten (*Martes flavigula*)				88	88
Leopard cat (*Prionailurus bengalensis euptilurua*)			6	86	92
Manchurian hare (*Lepus mandshuricus*)				276	276
Asian badger (*Meles leucurus*)				1187	1187

## Data Availability

The datasets generated and/or analyzed during the current study are available from the corresponding author upon reasonable request.

## Supplementary Materials

The following are available online at https://www.mdpi.com/article/10.3390/ani12111392/s1, Figure S1: Map of our camera-trapping study area in Northeast China, adjacent to the Land of Leopard National Park in Russia, Figure S2: The geographical distribution of the Chinese water deer *Hydropotes inermis inermis* before the 1990s according to Zhang (1997), Figure S3: Maximum likelihood tree using cytochrome *b* sequences from deer species, Table S1: Water deer sample information for genetic analysis.
