# Supplementary material for: Northward Range Expansion of Water Deer in Northeast Asia: Direct Evidence and Management Implications
Source: Animals (Basel). 2022 May 28;12(11):1392. doi: 10.3390/ani12111392 (PMC9179514; doi:10.3390/ani12111392)
Supplement: Supplementary file 1 [file animals-12-01392-s001.zip › animals-1688740-supplementary.pdf]

SUPPLEMENTARY MATERIALS

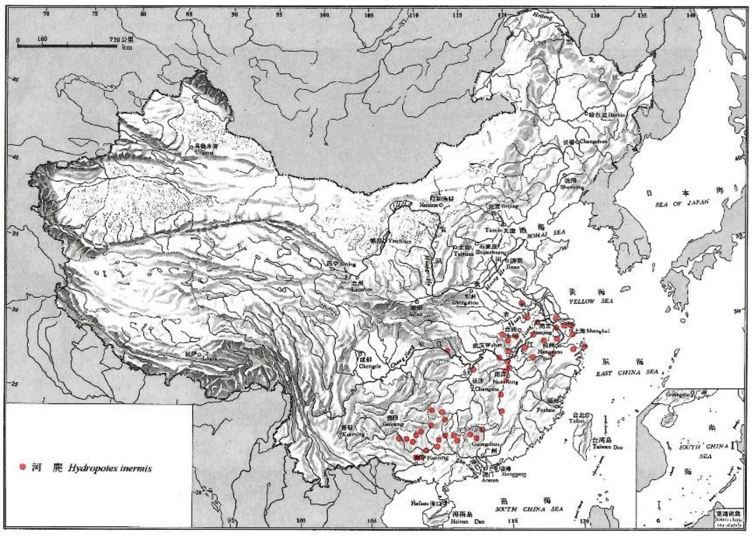

Figure S1. The geographical distribution of the Chinese water deer *Hydropotes inermis inermis* before 1990s according to Zhang (1997). Red points approximately mark regions of occurrences. Data were collected from the following sources: (1)Early publications, of which the most important are: N. Kuroda(1940), *Picture Album of Japanese Mammals*; AlIen, G. M, *The Mammals of China and Mongolia* (1938-1940); EIlerman, J. R. and J. C. S. Morrison-Scott (1950), *Checklist of Palaearctic and Indian Mammals*; Loukashkin, A. S (1937), *Wild Mammals of Northern Manchuria*; Bobrinskii, N. A., B. A. Kuznechov and A. P. Kuzakin (1944), *Identification of Mammals of U. S. S. R*; Ognev, S. E. (1928-1950), *Beasts of U. S. S. R. and Its Adjacent Countries* (Vol. 1-7), ed. Shou Zhenhaung (1958) , *Survey Report of Mammals in Northeast China chief*; Shou Zhenhuang (1962), *Fauna Economica of China-Mammalia*; Qian Yanwen *et al* (1965), *The Birds and Beasts of Southern Xinjiang; Mammals*. (2) Information on faunal investigations and records of specimens, contributed by integrated scientific expeditions of Academia Sinica since the 1950s; (3) Publications about mammals at provincial level for the past two decades: Ma Yiqing (chief ed.) (1986), *Fauna of Heilongjiang-Mammalia*; Xiao Zhenggu (chief ed.) (1988), *Fauna of Liaoning-Mammals*; Wang Qishan (chief ed.) (1990), *Mammal Fauna of Anhui*; Zhuge Yang (chief ed.) (1988), *Fauna of Zhejiang-Mammalia*; Hu Jinzhu and Wang Youzi (1982, 1984), *Sichuan Fauna Economica-Mammalia*; Luo Ren (chief ed.) (1993), *The Mammals of Guizhou*; Sheng Lantian et al. (1988), *Vertebrate List of Guangxi-Mammalia*; Feng Zuojian et al (1986), *The mammals of Xizang*; Xu Longhui et al (1983), *The Birds and Beasts of Hainan*; Li Dehao et al (1989), *The Economic Vertebrates of Qinghai*; Wang Xiantin (chief ed.) (1991), *Vertebrate Fauna of Gansu*; Wang Shibo et al (1983), *Rodentia Fauna of Xinjiang*; Ma Yong et al (1987), *Glires (Rodents and Lagomorphs of Northern Xinjiang and Their Zoogeographical Distribution*; Wang Tingzheng and Xu Wenxian (chief ed.) (1992) , *Glires (Rodentia and Lagomorpha) Fauna of Shaanxi Province*; Gao Yaoting et al. (1987) , *Fauna Sinica: Mammalia-Vol. 8:*

Carnivora; Xu Yongchun et al (1987); *Integrated Scientific Report of Xishuangbanna Nature Reserves*; Yin Binggao et al. (1991), *Wildlife Protection in Tibet*; Wu Zhengyi et al. (1983), *Research of Forest Ecosystems on Ailao Mountains, Yunnan*.

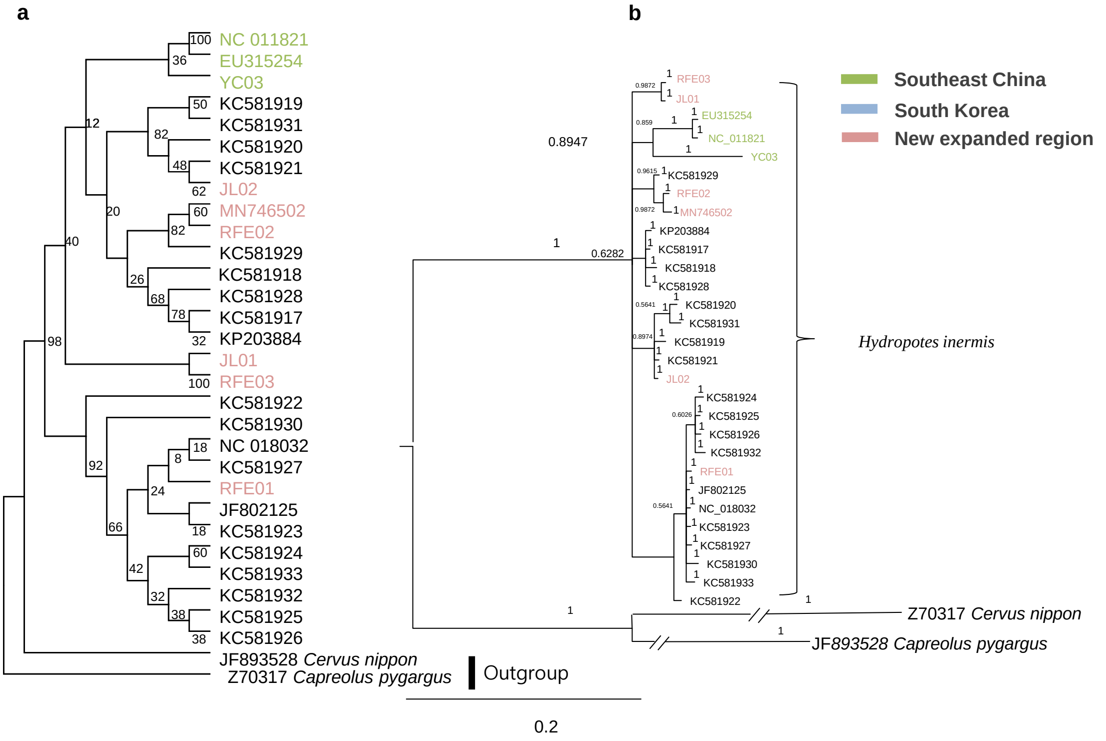

Figure S2. Maximum likelihood and Bayesian inference trees based on control region data from water deer and two outgroups (a: Maximum likelihood tree; b: Bayesian inference tree). The jModelTest v 2.1.7 (Guindon and Gascuel, 2003; Darriba et al., 2012) provided the best fit substitution model for each phylogenetic tree reconstruction. Independent phylogenetic trees were constructed for cytochrome b and D-loop sequences. Software MEGA was used to construct the maximum likelihood phylogenetic tree with 1,000 bootstraps (Tamura et al., 2011). The Bayesian Inference(BI) tree was constructed using the MrBayes v 3.2.7 (Ronquist et al., 2012). Siberian roe deer (Capreolus pygargus) and sika deer (Cervus nippon) were used as outgroups (GenBank accession no. Z70317; JF893528) during phylogenetic analysis.

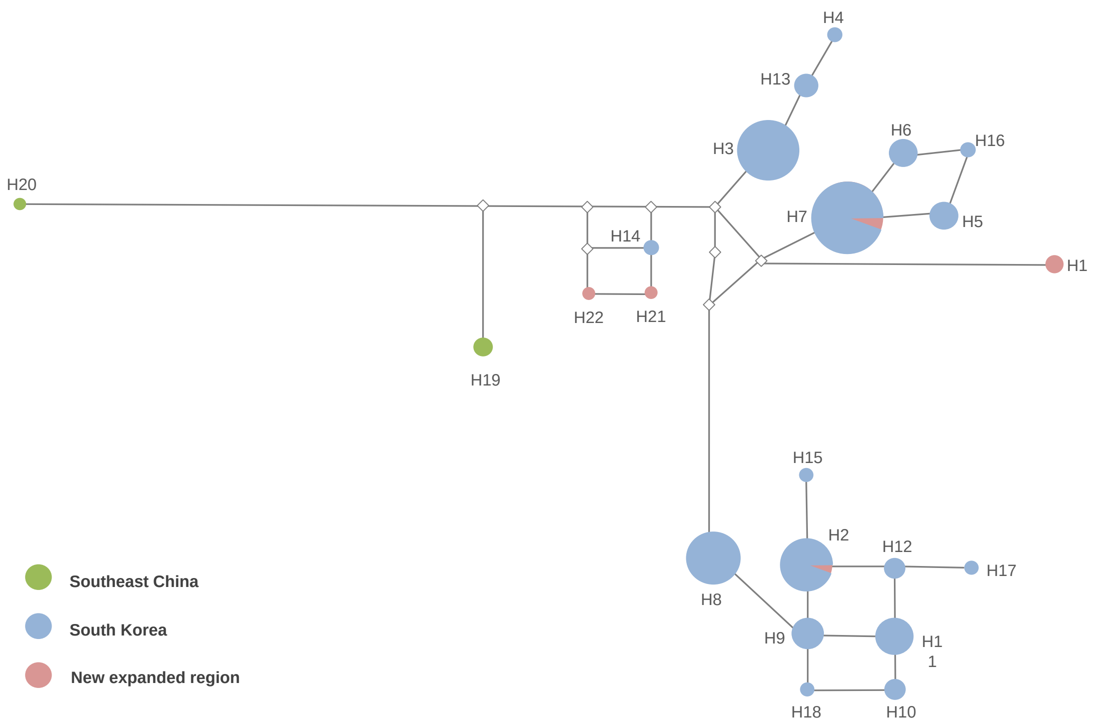

Figure S3. Median-joining network (MJN) analysis with control region haplotypes of water deer. median-joining network (MJN) of water deer haplotypes (D-loop sequence) using the program Network v 10 (Bandelt et al., 1999) to visualize the relationship among populations.

Table S1. Water deer sample information for genetic analysis.

Sample ID	Area	Location	Sample type	Date	Notes
JL01	Bolidong, Hunchun, Jilin Province, China	E 130.44102, N 42.55645	Tissue	2019-7	Roadkill
JL02	Baliancheng, Hunchun, Jilin Province, China	E 130.26988, N 42.87993	Tissue	2019-5	Roadkill
RFE01	Khasansky district of Primorsky Krai, Russia	E 130.47771, N 42.33729	Tissue	2019-12-11	Hunted as roe deer
RFE02	Khasansky district of Primorsky Krai, Russia	E 130.43504, N 42.34924	Tissue	2019-12-25	Hunted as roe deer
RFE03	Khasansky district of Primorsky Krai,Russia	E 130.65602, N 42.41273	Tissue	2020-2-15	Confiscated from poaching
